# The complete mitochondrial genome of *Poraniopsis inflata* (Asteroidea: Valvatida: Poraniidae) from Dokdo Island, Korea

**DOI:** 10.1080/23802359.2024.2317321

**Published:** 2024-02-19

**Authors:** Maria Alboasud, Hoon Jeong, Taekjun Lee

**Affiliations:** a Marine Biological Resource Institute, Sahmyook University, Seoul, Republic of Korea; b Department of Convergence Science, Sahmyook University, Seoul, Republic of Korea; c Department of Animal Resources Science, Sahmyook University, Seoul, Republic of Korea

**Keywords:** sea star, asteroids, mitogenome, phylogeny, taxonomy

## Abstract

This study presents the complete mitochondrial genome sequence of *Poraniopsis inflata*, providing valuable information on its genetic and taxonomic studies. Through next-generation sequencing, we successfully obtained the complete mitogenome of *P. inflata*, spanning a length of 16,322 bp. This genome structure encompasses 13 protein-coding genes (PCGs), 22 transfer RNA genes, and two ribosomal RNA genes. The phylogenetic analysis, based on a dataset of 13 PCG sequences, illuminated the phylogenetic relationships of *P. inflata* with other species of class Asteroidea and a species of echinoderm classes. The maximum likelihood phylogenetic tree showed that *P. inflata* closely clustered with *Linckia laevigata.* By revealing its mitochondrial genome and positioning it within the Asteroidea lineage, this study provides insights into the phylogenetic context of *P. inflata*.

## 1. Introduction

*Poraniopsis inflata* (Fisher, 1906) belongs to the family Poraniidae Perrier, 1894, within the order Valvatida Perrier, 1884, in the class Asteroidea de Blainville, 1830 (Mah 2023). This species was originally presented as *Alexandraster inflatus* by Fisher (1906) and subsequently transferred to the genus *Poraniopsis* Perrier, 1891. Lastly to *Poraniopsis inflata* by Fisher (1910). This species is widely distributed across the North Pacific region, extending from the adjacent waters of Korea to the states of Oregon and California on the northwest coast of America (Shin 2010). This study presents the first complete mitochondrial genome sequence of *P. inflata* based on 13 protein-coding genes (PCGs). This species exhibits distinct morphological characteristics, as outline below: (1) a body covered with a thick membrane and (2) prominent spines positioned at corners of papular areas.

## 2. Materials and methods

**Figure 1.**
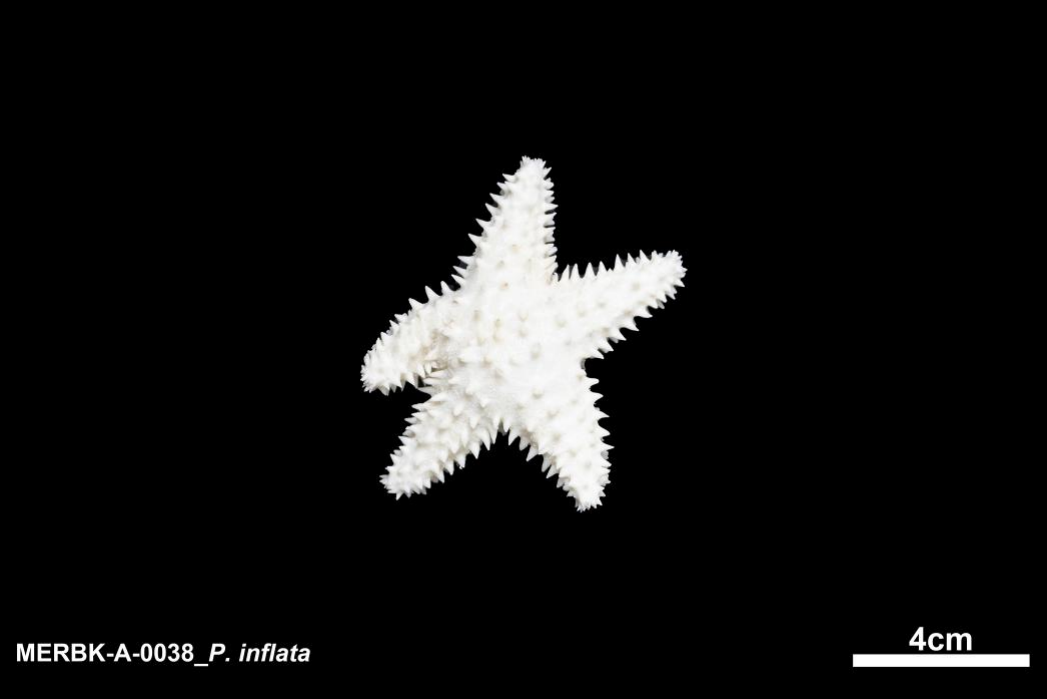
Species reference image of *Poraniopsis inflata* collected from Dokdo Island, Korea. Photographed by Taekjun Lee. This specimen in an ethyl alcohol solution (>95%) immediately after collected.

A living sample of *P. inflata* in this study was collected by Taekjun Lee, through trimix diving at a depth of 45.5 m, from Dokdo Island (37°14′52.8″N 131°52′01.2″E) on October 14, 2022, and was immediately preserved in ethyl alcohol solution (>95%) (Figure 1). A specimen was deposited at the Marine Echinoderm Resources Bank of Korea (MERBK) (https://biobank.mbris.kr, Taekjun Lee, leetj@syu.ac.kr) in Sahmyook University under the voucher number MERBK-A0038.

The total genomic DNA of *P. inflata* was isolated from ethanol-preserved gonad tissues using a DNeasy blood and tissue DNA isolation kit (Qiagen, Hilden, Germany) according to the manufacturer’s instructions. Genomic DNA quality and concentration were determined using a Nanodrop One-C spectrophotometer (Thermo Fisher Scientific, Waltham, MA, USA). The mitochondrial DNA was amplified using the REPLI-g Mitochondrial DNA Kit (Qiagen, Hilden, Germany) following the manufacturer’s protocol.

Next-generation sequencing (NGS) analysis was performed using genome analysis units at the National Instrumentation Center for Environmental Management at Seoul National University in Korea. A genomic library was constructed from the genomic DNA using a Kapa Hyper Prep Kit (Kapa Biosystems, Woburn, MA, USA), using paired-end reading, which was followed by NGS on the Illumina Hi-Seq 2500 platform (San Diego, CA, USA). The contigs of the mitogenome were assembled using the de novo assembly method on Geneious Prime 2023.1.1 (Biomatters Ltd, Auckland, New Zealand). The average depth of coverage is shown in Supplementary Figure 1. Transfer RNA (tRNA) genes were identified using tRNAscan-SE online (Lowe and Chan 2016), with the following search mode: the sequence source was ‘other mitochondrial’ and the genetic code for tRNA isotype prediction was ‘Invertebrate Mito’.

Mitogenome sequences were aligned using MAFFT (Katoh and Standley 2013) and the nucleotide dataset of 13 protein-coding genes (PCGs) was analyzed using maximum likelihood (ML) with RAxML 8.2 (Stamatakis 2014). The best-fit substitution was estimated using jModelTest 2.1.1 (Guindon and Gascuel 2003; Darriba et al. 2012) for the nucleotide dataset of 13 PCGs, and the selected substitution model for this study was GTR + I + G. This dataset encompassed 21 asteroids, including *P. inflata*, and two crinoid, two ophiuroids, two echinoids, two holothuroid, and two hemichordates as outgroup species. For ML analysis, the bootstrap resampling with 1000 iterations was conducted using the rapid option.

## 3. Results

**Figure 2.**
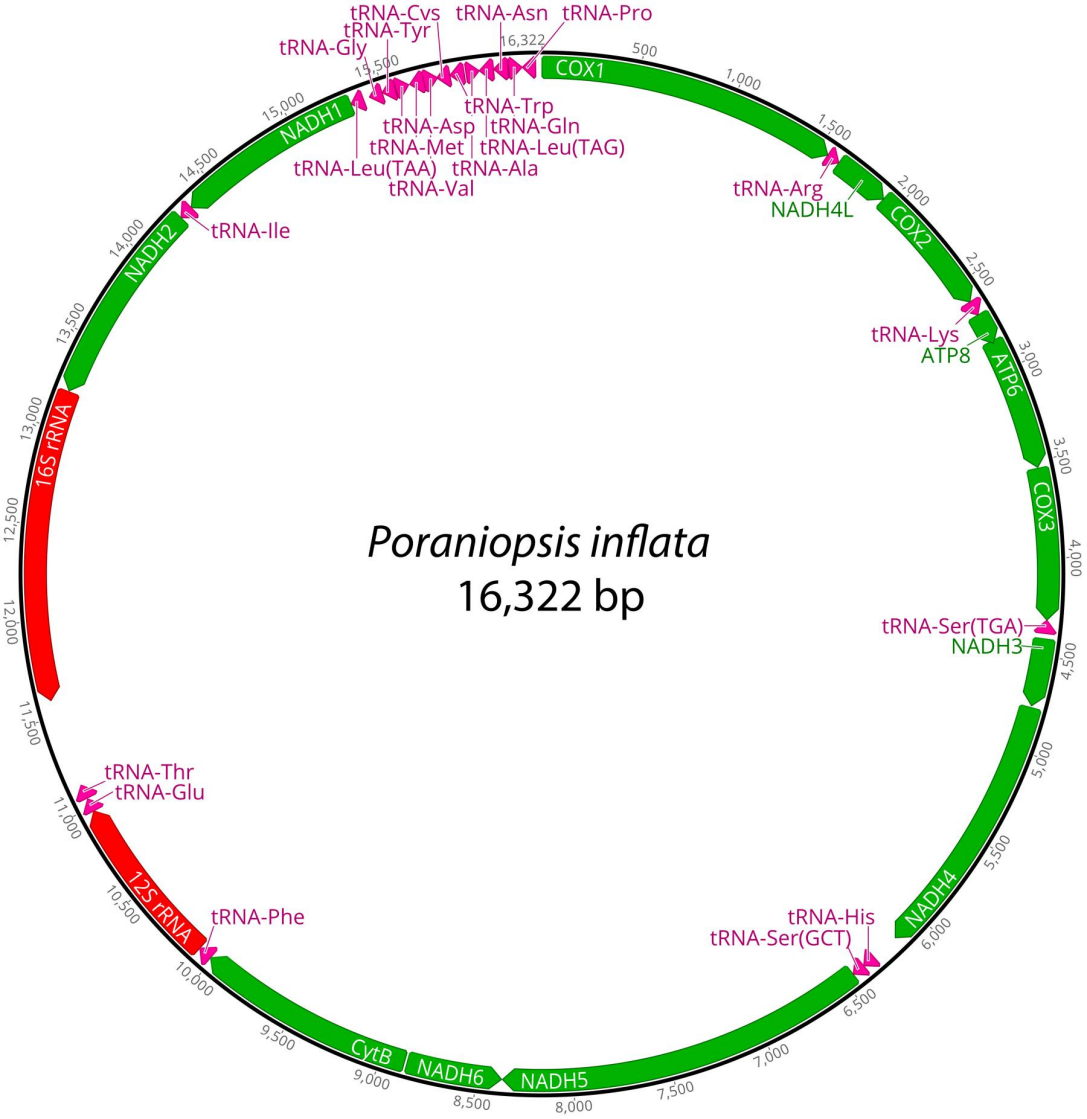
The complete mitochondrial genome of *Poraniopsis inflata* in this study. This map was generated by Geneious Prime ver. 2023.2.1.

**Figure 3.**
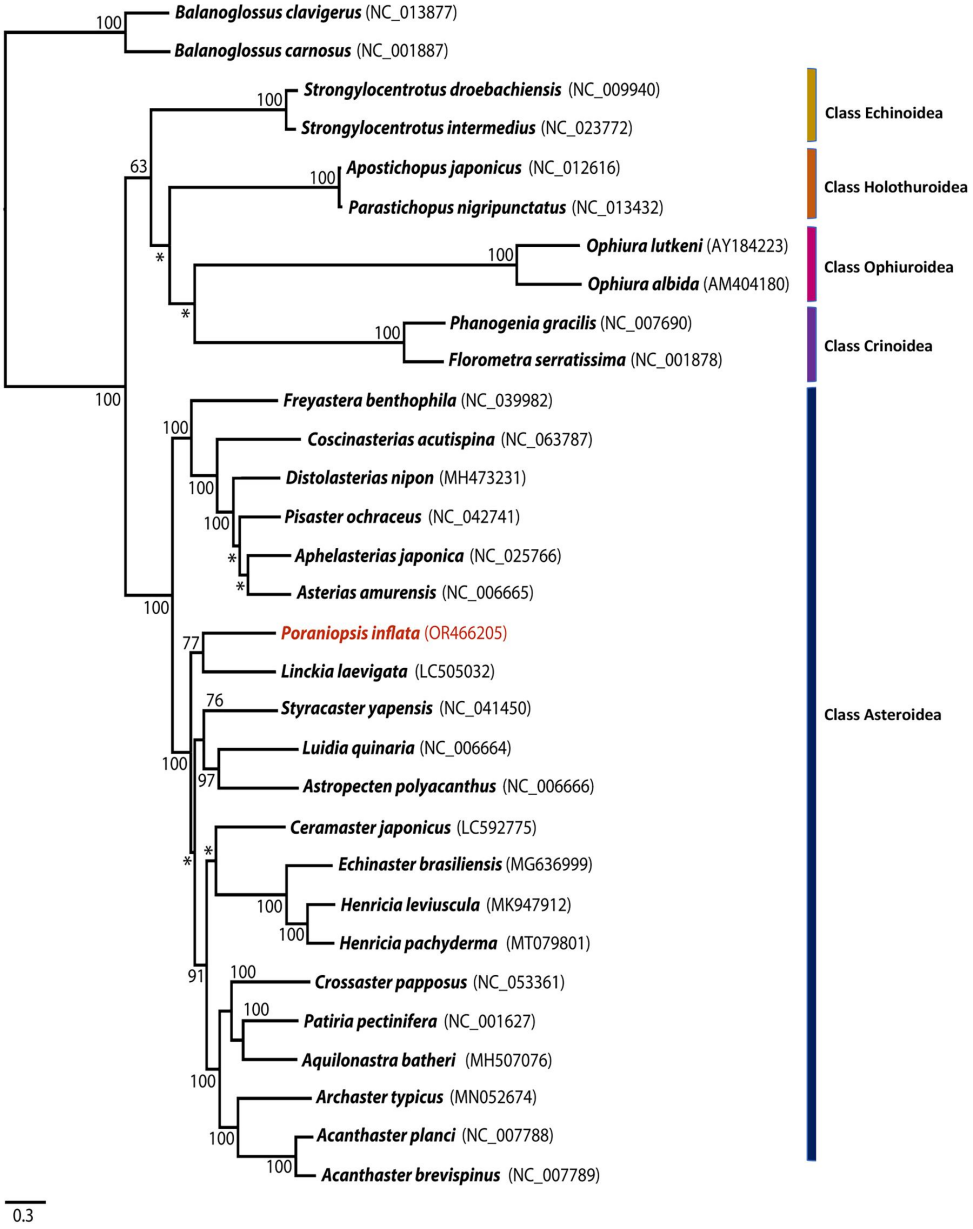
Phylogenetic analysis of *Poraniopsis inflata* and an additional 28 echinoderms was conducted using the maximum likelihood method based on the nucleotide sequences of 13 protein-coding genes. Two hemichordates, *balanoglossus carnosus* and *B. clavigerus*, were used as outgroups. The bootstrap support values are presented on each node, with values exceeding 50, while the asterisk marks indicate values below 50. Genbank accession numbers for published sequences are incorporated.

The mitogenome of *P. inflata* was 16,322 bp in length and contained 13 PCGs, 22 tRNA genes, and two ribosomal RNA (rRNA) genes (Figure 2). The base composition analysis of *P. inflata* revealed the following distribution: A (31.6%), T (29.5%), G (14.2%), and C (24.6%). Notably, the complete mitochondrial genome of *P. inflata* exhibited a slight anti-G bias (−0.2) and a preference for GC content (38.8%). The PCGs in the mitochondrial genome of *P. inflata* covered a total of 5,441 bp. Among them, NADH5 constituted the longest PCG, at 1923 bp, while ATP8 was the shortest, at 168 bp. The start codon ‘TAC’ was used for NADH1, NADH2, and NADH6, whereas the other PCGs initiated with the ‘ATG’ codon. The prevalent stop codon across various PCGs was ‘TAA’, employed by NADH1, NADH2, NADH4, NADH5, NADH6, ATP8, and ATP6. Meanwhile, COX1, COX2, CytB, NADH3, and NADH4L utilized the ‘TGA’ codon, and COX3 made use of ‘TAG’ as its stop codon. The ML phylogenetic tree based on 13 PCGs indicated that *P. inflata* formed a distinct monophyletic group alongside another species within the class Asteroidea, separated from other classes of the phylum Echinodermata (Figure 3). Within this clade, *P. inflata* exhibited monophyly with *Linckia laevigata* (Linnaeus, 1758), belonging to the same order Valvatida even though not the same family. The bootstrap support value was 77% (Figure 3).

## 4. Discussion and conclusion

Mitochondrial DNA (mtDNA) plays a pivotal role in elucidating phylogenetic relationships and understanding evolutionary biology, while also enabling the tracing of relationships between populations (Boore 1999). Thus, we present the first complete mitochondrial genome of *P. inflata* (GenBank accession number OR466205) in this study, aiming to expand our understanding of the phylogenetic relationships within the class Asteroidea.

In a previous study by Lee and Shin (2018), the complete mitochondrial genome of *Aquilonastra batheri* (Asteroidea, Valvatida) was examined, revealing a genome size of 16,463 bp, housing 13 PCGs, 22 tRNA genes, and two rRNA genes. Similarly, the mitochondrial genome of *P. inflata* in this study spans 16,322 bp, encompassing 13 PCGs, 22 tRNA genes, and two rRNA genes. Comparative analysis indicates that the mitochondrial genome structure of *P. inflata* closely resembles that of most previously studied asteroids.

Phylogenetic analysis is a valuable tool for identifying biological characteristics, studying species relationships, and exploring evolutionary history (Rubinoff and Holland 2005). Sun et al. (2022) conducted a study on the molecular phylogeny of 30 asteroid species. Through a phylogenetic analysis based on 13 PCGs using ML, it was observed that *Crossaster papposus*, which belonging to order Valvatida, formed a monophyletic clade with a robust bootstrap support value of 100%. Additionally, a larger clade comprising species from the order Valvatida displayed bootstrap support values ranging from 70% to 100% (Sun et al. 2022).

Remarkably, the phylogenetic analysis revealed that *P. inflata* forms a monophyletic group with *Linckia laevigata*, both belonging to the order Valvatida, despite not sharing the same family (Figure 1). This suggests that the order Valvatida stands out as a monophyletic clade distinct from the remaining orders within the class Asteroidea. These findings contribute significantly to our understanding of the phylogenetic relationships of *P. inflata* and provide valuable insights into the evolutionary history of this species.

## Supplementary Material

Supplemental Material

Supplemental Material

## Data Availability

BioSample, BioProject, and SRA accession numbers are https://www.ncbi.nlm.nih.gov/biosample/?term=SAMN37526385, https://www.ncbi.nlm.nih.gov/bioproject/PRJNA1020671, and https://www.ncbi.nlm.nih.gov/sra/SRR26160490, respectively. The genome sequence data that support the findings of this study are openly available in GenBank of National Center for Biotechnology Information (NCBI) at https://www.ncbi.nlm.nih.gov, with an accession number OR466205.
