# Supplementary figures and images for: The complete mitochondrial genome of Poraniopsis inflata (Asteroidea: Valvatida: Poraniidae) from Dokdo Island, Korea
Source: Mitochondrial DNA B Resour. 2024 Feb 19;9(2):290–4. doi: 10.1080/23802359.2024.2317321 (PMC10878332; doi:10.1080/23802359.2024.2317321)

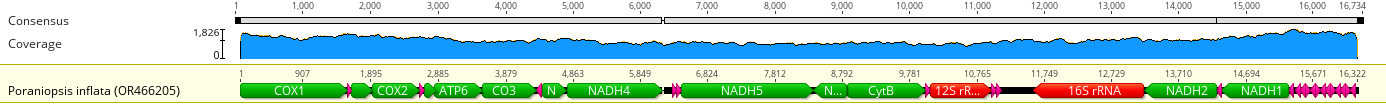

Supplement: Supplemental Material [file TMDN_A_2317321_SM9233.png]

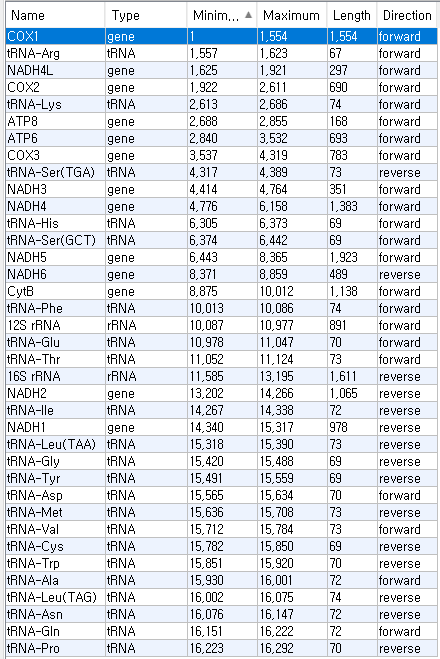

Supplement: Supplemental Material [file TMDN_A_2317321_SM9232.png]
